# Offene Latarjet-Patte-Operation nach Walch: Tipps, Tricks und Komplikationsvermeidung

**DOI:** 10.1007/s00113-022-01282-w

**Published:** 2023-02-02

**Authors:** S. Bauer, B. Dietz, P. Collin, L. Neyton, W. Blakeney, M. Zumstein

**Affiliations:** 1 Hôpital de Morges, Ensemble Hospitalier de la Côte, Chemin du Crêt 2, 1110 Morges, Schweiz; 2 St. Josefs-Hospital, Wiesbaden, Deutschland; 3 Clinique Victor Hugo, Paris, Frankreich; 4 Centre Orthopédique Santy, Lyon, Frankreich; 5 Royal Perth Hospital, Perth, Australien; 6 Inselspital und Orthopädie Sonnenhof, Bern, Schweiz; 7 School of Surgery, University of Western Australia, Perth, Australia

## Einleitung

Walch hat die Latarjet-Patte-Operation in offener Technik unter Anwendung eines Subskapularis-Splits in den 1980er-Jahren modifiziert, standardisiert und über Jahrzehnte mit großem Erfolg bei über 3500 Patienten angewandt. In Langzeitnachuntersuchungen wurden Rezidivraten von 1–5 % bei hoher Patientenzufriedenheit im Subjective Shoulder Value (SSV > 90 %) dokumentiert [1, 2]. Diese Ergebnisse konnten in Zürich durch Gerber reproduziert werden [3].

Die Methode hat in mehreren Langzeitstudien den Beweis des Erreichens dauerhafter Stabilität (Langzeitstabilität nach mehr als 6 bis 10 Jahren) bei Risikopatienten erbracht [2, 3], besonders für Wettkampfsportler [4–6], junge Patienten [7, 8] und Patienten mit glenoidalem Knochenverlust [1, 9]. Bei korrekter Operationstechnik entsteht kein klinisch relevanter Beweglichkeitsverlust, und es besteht kein Zusammenhang mit der Entstehung oder Progression einer Instabilitätsarthrose [10]. In einer randomisierten kontrollierten Studie (RCT) zeigte sich eine signifikant niedrigere Reluxationsrate für die Latarjet-Stabilisierung bei jungen Männern unter 25 Jahren im Vergleich zur arthroskopischen Bankart-Operation [8].

## Definition

**Figure 1:**
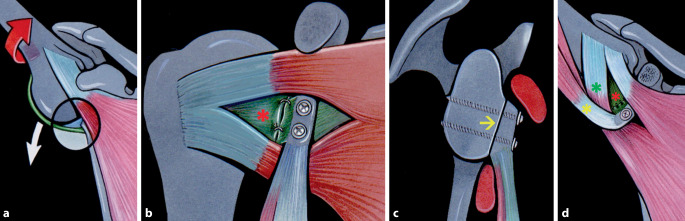

Bei der Latarjet-Patte-Technik nach Walch wird der Processus coracoideus unter Erhalt einer Länge von durchschnittlich 24 mm osteotomiert, danach abgeflacht und vorgebohrt. Es erfolgen ein muskulärer Subskapularis-Split im Verhältnis zwei Drittel oben zu einem Drittel unten bei angelegtem Arm in Außenrotation vor einer vertikalen, möglichst medialen Arthrotomie parallel zur Gelenkfläche, Knochenanfrischung, freihändige Positionslochbohrung und Rotationsfeinjustierung des flach platzierten Knochenblockes, der mit zwei 4,0-mm- oder 4,5-mm-Teilgewinde-Sponiosa-Schrauben fixiert wird. Auf sonstige Bohr- und Justierungszangen und kanülierte Schrauben wird verzichtet. Die Stabilisierung wird durch 4 Effekte erreicht (Abb. 1):

1. Knochenblock,
2. Schlingeneffekt (Conjoint-Sehne),
3. Hängematteneffekt (unterer Subskapularis wird distalisiert),
4. Vernähen des korakoakromialen Ligamentstumpfes (CAL) mit der Kapsel.

## Indikation

Die Indikation sollte bei chronisch traumatischer, anteriorer Schulterinstabilität mit Dokumentation (Röntgendokumentation) mit oder ohne Hyperlaxizität und Nachweis einer kapsuloligamentären Läsion gestellt werden, oder in Einzelfällen bei Erstluxation aufgrund des Risikoprofils. Kontraindikationen sind willkürliche Luxationen ohne traumatische Läsion, multidirektionelle Instabilität sowie Luxationen und Subluxationen bei der Mehrzahl älterer Patienten (> 50 Jahre) [11]. Mithilfe des Instabilität-Schwere-Index-Scores (ISIS) kann das Rezidivrisiko bei alleiniger Weichteilstabilisierung eingeschätzt werden [4, 5].

## Lagerung

**Figure 2:**
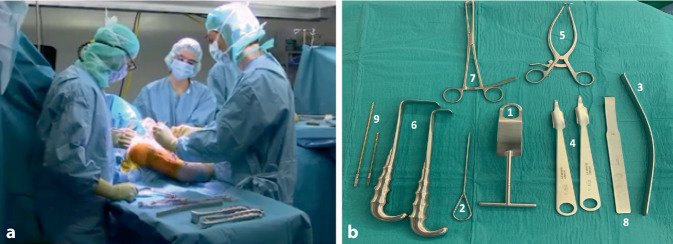

Die 45°- bis 60°-halbsitzende Lagerung mit Unterlage eines Tuches unter den medialen Skapularand bei frei beweglichem Arm ohne Armhalter hat sich bewährt. Die Ecke eines höhenverstellbaren Mayo-Tisches wird zwischen erstem Assistenten und Operateur zur Armlagerung eingesetzt. Ein zweiter Assistent steht auf der gegenüberliegenden Seite des Patienten (Abb. 2).

## Hebel und Retraktoren

**Figure 3:**
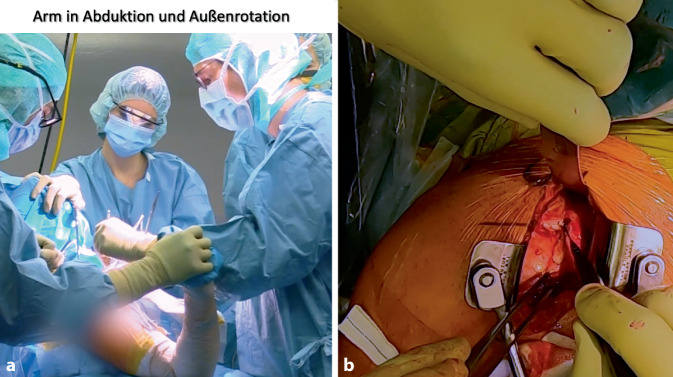

Um eine gute 360°-Darstellung des Situs zu erhalten, haben sich ein glatter, stumpfer Fukuda- oder Trillat-Hebel ohne Kanten zum transartikulären Einsatz (Abb. 2b, Nr. *1*), ein auf den letzten 6–10 cm gerader Link-Hebel („Batman“, Nr. *3*), ein Standard-Hohmann-Hebel (Nr. *4*) und ein Rocher-Nagel (Nr. *2*) mit Handgriff bewährt. Des Weiteren kommen Gelpi- und Weitlaner-Spreizer ebenso wie der Richardson-Schulterhaken (Nr. *6*) zum Einsatz (Abb. 3).

## Inzision und oberflächliche Dissektion

Die standardisierte Technik nach Walch wurde als Ausbildungsvideo frei zugänglich veröffentlicht [12]. Unterhalb des Processus coracoideus, vertikal entlang der Langer-Linien, wird die kosmetisch gut heilende Inzision angelegt („BH-Trägerschnitt“ mit einer Länge von 6–8 cm). Im oberen deltopektoralen Intervall wird die Mohrenheim-Grube aufgesucht. Die Dissektion wird medial am Pektoralis gehalten. um die V. cephalica schonend nach lateral zu mobilisieren.

## Schlüsselmanöver und Schlüsseletappen

Zur Standardisierung der Technik wurde *3 Schlüsselmanöver* beschrieben:

(1) Armpositionen, (2) Handhabung von Hebeln und Retraktoren und (3) sicheres Release der Conjoint-Sehne.

Des Weiteren *6 Schlüsseletappen*:

(1) Korakoideus-Exposition und Primär-Release, (2) Osteotomie und weiteres Release, (3) Knochenblockpräparation, (4) Subskapularis-Split und Arthrotomie, (5) 360°-Skapulahals-Exposition und (6) Cornerstone-Bohrloch-Positionierung, Fixierung und einfache Kapsuloplastie.

## Tipps und Tricks

**Figure 4:**
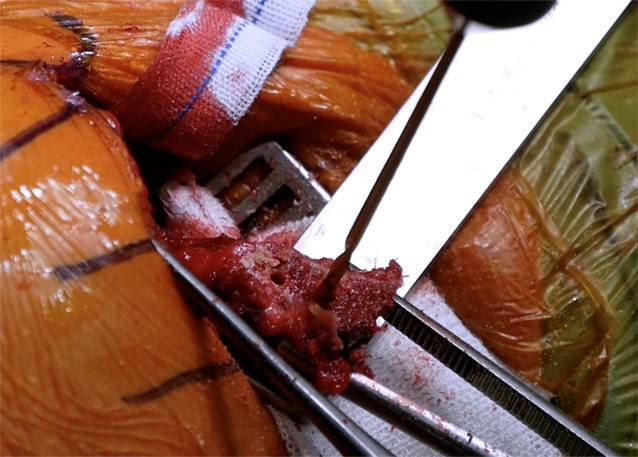

**Figure 5:**
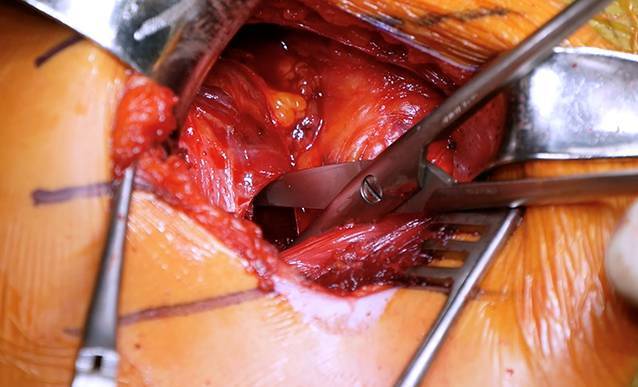

**Figure 6:**
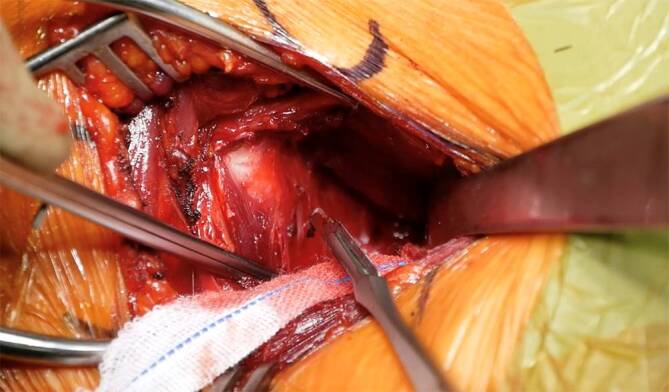

**Figure 7:**
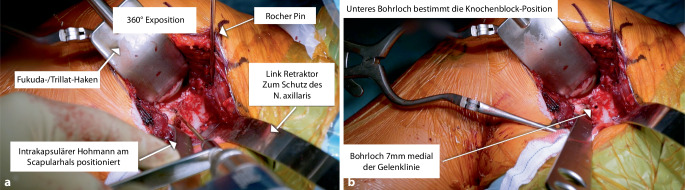

**Figure 8:**
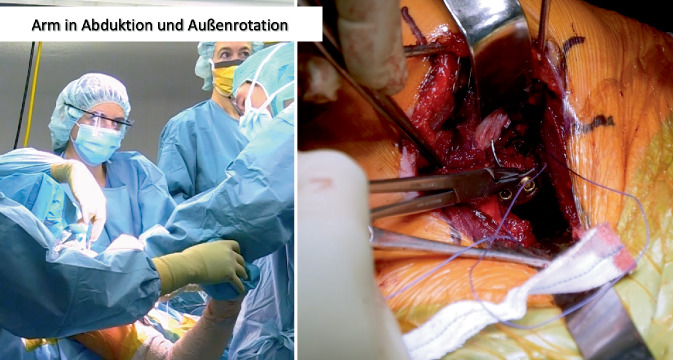

Sequenz der Armpositionen und Tipps:

1. *Abduktion* *+* *Außenrotation*: Release des lateralen Processus coracoideus, Durchtrennung des CAL (Abb. 3)
2. *Adduktion* *+* *Innenrotation*: Release des M. pectoralis minor, Osteotomie: medial nach lateral
3. *Abduktion* *+* *Außenrotation*: Posteriores und laterales Conjoint-Sehnenrelease, *Cave:* Kein mediales Release distal der Korakoideus-Spitze!
  *Tipp*: Leichte Traktion hilft bei Schwierigkeiten das korakohumerale Ligament (CHL) zu durchtrennen
4. *Neutralposition*: Korakoideus evertieren, abflachen, anfrischen und vorbohren (Abb. 4)
5. *Adduktion (angelegter Arm)* *+* *Außenrotation*: Darstellung des muskulären Subskapularis: Split durch vertikales Spreizen der Schere (Abb. 5), *Cave:* vor horizontalem Spreizen Axillaris und Plexus durch Hebel schützen!!
  *Tipp: *Einlage einer gestielten Kompresse in die Fossa subscapularis vor Einsetzen des Hebels
  *Tipp: *Freischaben der weißen Kapsel mittels Raspatorium, dann Einsatz des Gelpi-Spreizers
  *Tipp:* vertikale Kapsulotomie immer von medial nach lateral und möglichst weit medial, parallel zum Gelenk *mit frischer Klinge *für eine ausreichende Länge des lateralen „Kapselvorhangs“ (Abb. 6)!
  *Tipp:* 360°-Exposition mit 4 Hebeln zur Knochenblockpositionierung (Abb. 7)
  *Tipp:* Das untere Bohrloch bestimmt die Position vor Rotationsjustierung! Cave: kein lateraler Überhang!
6. *Abduktion* *+* *Außenrotation:* Naht des CAL-Stumpfes an die Kapsel („Kapselvorhang“) in 45°-Außenrotation (Abb. 8).

## Komplikationsvermeidung

**Figure 9:**
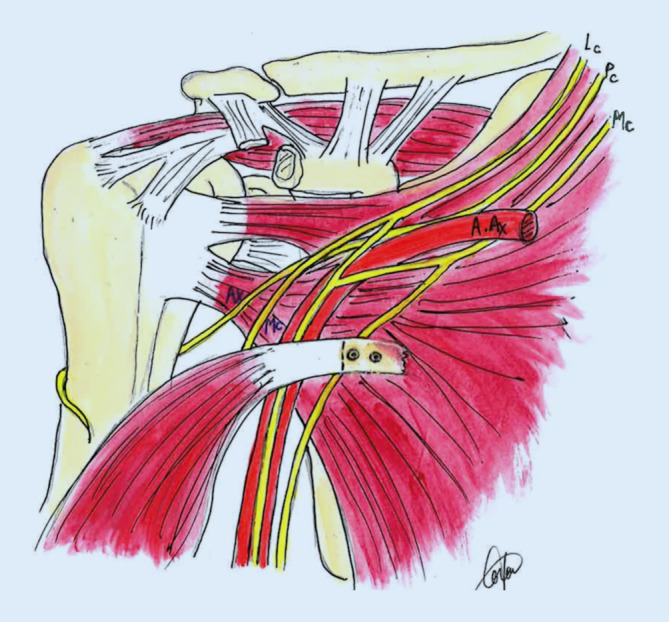

**Figure 10:**
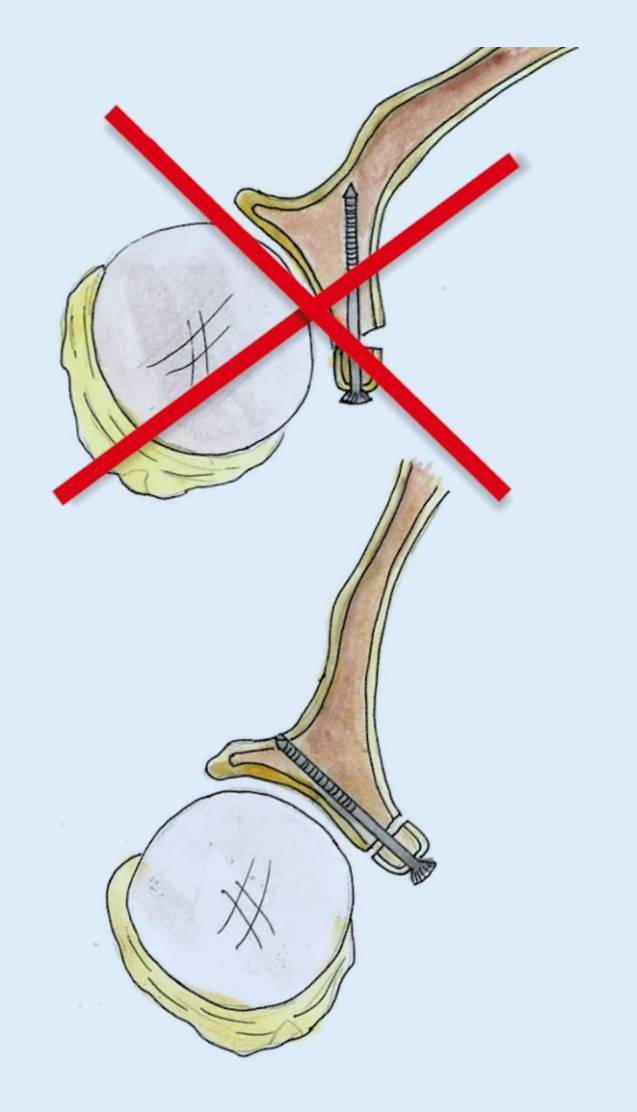

1. Zu kurzer Korakoideus nach Osteotomie (mindestens 22–25 mm): vorher messen!
2. Mediales Release: *Cave*: Verletzung des N. musculocutaneus: Stopp an medialer Korakoidspitze!
3. Zu starke Anfrischung der Knochenblockunterseite, zu enge Bohrung: *Cave*: Fraktur! Kortexbrücke zwischen Löchern belassen!
4. *Cave*: Verletzung des N. axillaris und Plexus (Abb. 9)! Einsatz eines Hebels (Link-Hebel, „Batman“) in die Fossa des Subskapularis vor horizontalem Split und Bohrungen am Glenoid!
5. *Cave*: Fehlpositionierung des Knochenblockes: Position des unteren Bohrloches, 7 mm medial des Gelenkspaltes und Bohrrichtung parallel zum Gelenkspalt! (Abb. 10)
6. Cave: postoperative Hämatome: Einlage einer Drainage!

## Nachbehandlung

Frühzeitige Übungen in geschlossener Kette und zur Beweglichkeit mit Belastungsaufbau nach 6 bis 8 Wochen und Wiederaufnahme von Wettkampfsport nach 3 bis 5 Monaten.
